# Context-Dependent Modulation of Excitatory Synaptic Strength by Synaptically Released Zinc

**DOI:** 10.1523/ENEURO.0011-17.2017

**Published:** 2017-03-03

**Authors:** Bopanna I. Kalappa, Thanos Tzounopoulos

**Affiliations:** Departments of Otolaryngology and Neurobiology, University of Pittsburgh, Pittsburgh, PA 15261

**Keywords:** auditory brainstem, auditory synapses, short-term plasticity, synaptic zinc, ZnT3

## Abstract

Synaptically released zinc inhibits baseline excitatory neurotransmission; however, the role of this neuromodulator on short-term plasticity during different levels of synaptic activity remains largely unknown. This lack of knowledge prevents our understanding of information transfer across zinc-releasing synapses, including 50% of excitatory synapses in cortical areas. We used *in vitro* electrophysiology in mouse brain slices and discovered that the effects of zinc on excitatory postsynaptic current (EPSC) amplitudes are context-dependent. At lower frequencies of activity, synaptically released zinc reduces EPSC amplitudes. In contrast, at higher stimulation frequencies and vesicular release probability (Pr), zinc inhibits EPSC amplitudes during the first few stimuli but leads to enhanced steady-state EPSC amplitudes during subsequent stimuli. This paradoxical enhancement is due to zinc-dependent potentiation of synaptic facilitation via the recruitment of endocannabinoid signaling. Together, these findings demonstrate that synaptically released zinc is a modulator of excitatory short-term plasticity, which shapes information transfer among excitatory synapses.

## Significance Statement

In many brain areas, including the neocortex, limbic structures, and auditory brainstem, glutamatergic nerve terminals contain zinc in their synaptic vesicles. Zinc is loaded into these vesicles by zinc transporter 3 and is coreleased with glutamate. Synaptically released zinc is an inhibitory neuromodulator in excitatory synapses, but the role of zinc in short-term plasticity remains unknown. Our results suggest that zinc shapes excitatory synaptic strength in a manner dependent on frequency and activity level. Namely, during low vesicular release probability (Pr) and low-frequency stimulation, zinc inhibits EPSCs; during higher Pr and prolonged presynaptic stimulation, zinc enhances steady-state EPSCs.

## Introduction

Zinc is an essential element for cellular function. Divalent zinc is a cofactor in a large number of enzymes and regulatory proteins (Vallee, 1988), and as such, the chemistry and biology of zinc metalloproteins have historically dominated the field of zinc biology. However, in recent years there has been growing evidence for a signaling role of mobile chelatable zinc, which is released in tissues such as the prostate, pancreas, and brain (Frederickson et al., 2005; Kelleher et al., 2011). In the brain, the vesicular zinc transporter, ZnT3, sequesters zinc into synaptic glutamatergic vesicles in many excitatory synapses. In the cortex, more than 50% of excitatory presynaptic terminals have vesicles that contain ZnT3, attesting to zinc’s importance in synaptic transmission (Sindreu et al., 2003; Frederickson et al., 2005). Recent studies used novel tools for chelating and tracking zinc in central synapses and established that zinc is a phasically released inhibitory neuromodulator in excitatory synapses. In response to a single presynaptic action potential, synaptic zinc is released from the presynaptic terminal and inhibits postsynaptic glutamate AMPA and NMDA receptor EPSCs (NMDARs/AMPARs) via postsynaptic mechanisms (Pan et al., 2011; Kalappa et al., 2015). Moreover, during brief repetitive synaptic stimulation, zinc inhibits synaptic and extrasynaptic glutamate NMDARs via postsynaptic mechanisms (Vergnano et al., 2014; Anderson et al., 2015) and is necessary—along with GPR39, a putative metabotropic zinc-sensing receptor—for activation of endocannabinoid signaling and inhibition of vesicular release probability (Pr; Perez-Rosello et al., 2013).

Zinc is released from glutamatergic vesicles and inhibits excitatory synaptic strength via pre- and postsynaptic mechanisms. Therefore, it is essential to study the mechanisms and dynamics of zinc modulation of excitatory neurotransmission during ongoing synaptic activity. The determination of these mechanisms will be crucial for understanding the role of synaptically released zinc in information transfer among excitatory synapses during different levels of synaptic activity. To study this issue, we combined electrophysiological, pharmacological, and genetic techniques.

## Materials and Methods

### Animals

In this study, we used male or female ICR mice (Harlan Laboratory) and ZnT3 wild-type (WT) and knockout (KO) littermate mice (Jackson Laboratory) aged from postnatal day 18 (P18) to P28. All animal procedures were approved by the Institutional Animal Care and Use Committee of the University of Pittsburgh, Pittsburgh, PA.

### Electrophysiology

**Figure 1.**
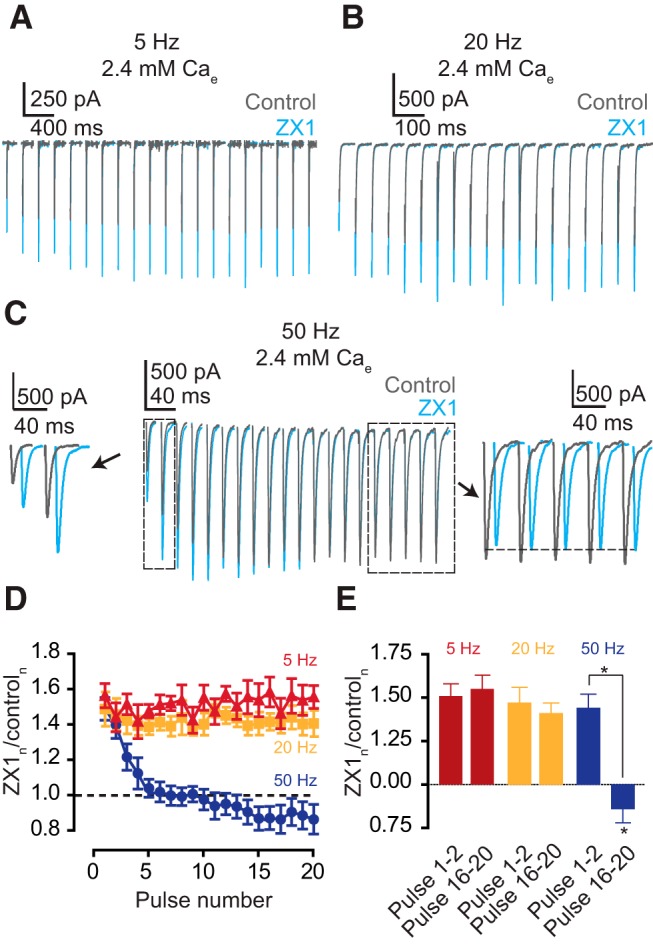
Frequency- and history-dependent effects of synaptically released zinc on AMPA EPSCs. ***A–C***, Representative traces of cartwheel cell AMPA EPSCs evoked by 20-pulse train stimulation of parallel fiber at 5 Hz (***A***), 20 Hz (***B***), and 50 Hz (***C*** middle panel), before (grey) and after 100 µM ZX1 (blue); (***C*** side panels) same as in upper panel but zoomed on the first 2 pulses (left) and the last 5 pulses (right). For improved visualization, responses after ZX1 are slightly shifted to the right. ***D***, Summary graph showing the ratio of AMPA EPSCs in ZX1 to that in control at each pulse at 5 Hz (maroon), 20 Hz (orange), and 50 Hz (blue). ***E***, Summary graph comparing the ratio of AMPA EPSCs in ZX1 to that in control for the first two and last five pulses in the 20-pulse train at 5 Hz (maroon, *n* = 10, *p* = 0.82), 20 Hz (orange, *n* = 10, *p* = 0.63), and 50 Hz (blue, *n* = 10, *p* < 0.01). Values represent mean ± SEM. Detailed values and statistical tests: ***D***, ***E***, ZX_1–2_/control_1–2_ vs. ZX_16–20_/control_16–20_: 5 Hz: 1.51 ± 0.07 vs. 1.55 ± 0.08, *n* = 10, *p* = 0.82, *t* = 0.24, df = 9; 20 Hz: 1.47 ± 0.09 vs. 1.41 ± 0.06, *n* = 10, *p* = 0.63, *t* = 0.49, df = 9; 50 Hz: 1.44 ± 0.07 vs. 0.86 ± 0.08, *n* = 10, *p* < 0.01, *t* = 5.45, df = 9; paired *t*-tests; ZX_1–2_/control_1–2_: 5 Hz vs. 20 Hz, *p* = 0.65, *t* = 0.46, df = 9; 5 Hz vs. 50 Hz, *p* = 0.38, *t* = 0.92, df = 9; 20 Hz vs. 50 Hz, *p* = 0.68, *t* = 0.42, df = 9; ZX_16–20_/control_16–20:_ 5 Hz vs. 20 Hz, *p* = 0.09, *t* = 1.87, df = 9; 5 Hz vs. 50 Hz, *p* < 0.01, *t* = 5.09, df = 9; 20 Hz vs 50 Hz, *p* < 0.01, *t* = 4.98, df = 9; paired *t*-tests; AMPA EPSC amplitudes_16–20_: control: 50 Hz: 1.00 ± 0.00 vs. ZX1: 0.86 ± 0.08, *p* = 0.042, *t* = 2.36, df = 9; one-sample *t*-test.

**Figure 2.**
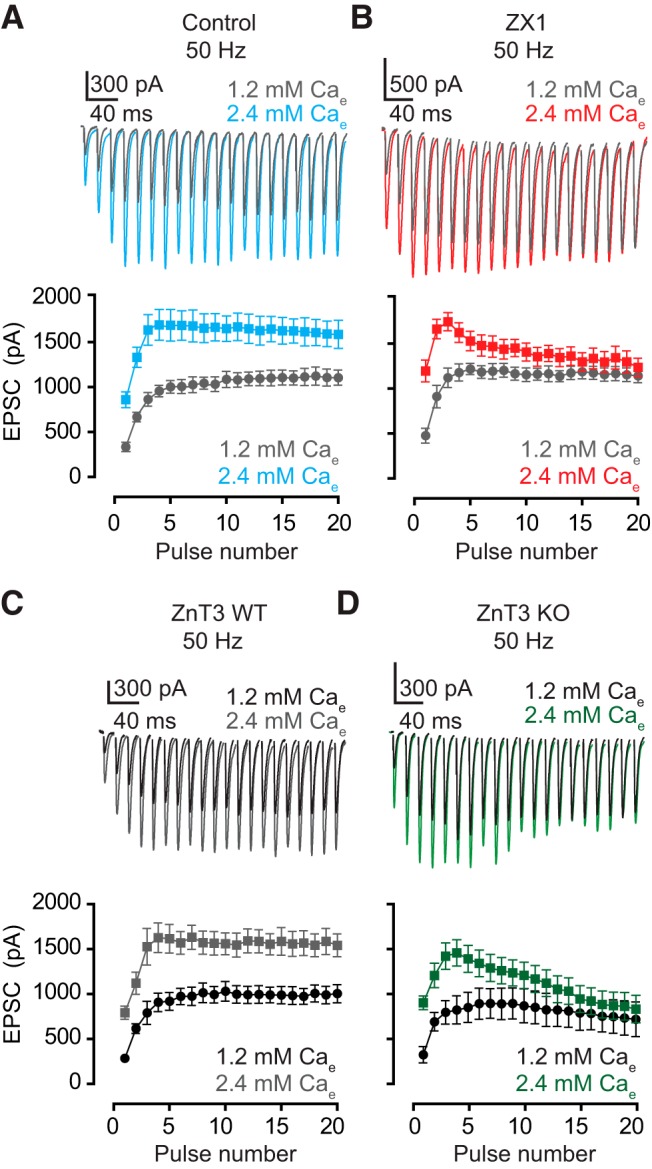
ZnT3-dependent, synaptically released zinc enhances EPSC_SS_ in high Pr. ***A***, ***B***, Upper panels, representative traces of AMPA EPSCs evoked by 50-Hz 20-pulse train in 1.2 mm (gray) and 2.4 mm (color) external calcium in control (***A***) and ZX1 (***B***). Lower panels, summary graphs showing average AMPA EPSC amplitudes during the train in 1.2 mm (gray) and 2.4 mm (color) external calcium in control (***A***) and ZX1 (***B***; EPSC_16–20_ amplitude: control: 1.2 vs. 2.4 mm: *n* = 7, *p*< 0.01; ZX1: 1.2 vs. 2.4 mm: *n* = 7, *p* = 0.09). ***C***, ***D***, Upper panels, representative traces of AMPA EPSCs evoked by 50-Hz 20-pulse train in 1.2 mm (black) and 2.4 mm (gray or green) external calcium in ZnT3WT (***C***) and ZnT3KO mice (***D***). Lower panels, summary graphs showing average AMPA EPSC amplitudes during the train in 1.2 mm (black) and 2.4 mm (gray or green) external calcium in ZnT3WT (***C***) and ZnT3KO mice (***D***; ZnT3 WT: 1.2 vs. 2.4 mm: *n* = 5, *p* < 0.01; ZnT3KO: 1.2 vs. 2.4 mm: *n* = 5, *p* = 0.24). Values represent mean ± SEM. Detailed values and statistical tests. ***A***, ***B***, lower panels: EPSC_16–20_ amplitude: control: 1.2 mm calcium: 1109 pA ± 90 pA vs. 2.4 mm calcium: 1605 pA ± 147 pA, *n* = 7, *p* < 0.01, *F* = 40.89, DFn = 1, DFd = 60; ZX1: 1.2 mm calcium: 1158 pA ± 75 pA vs. 2.4 mm calcium: 1301 pA ± 116 pA, *n* = 7, *p* = 0.09, *F* = 2.9, DFn = 1, DFd = 60; two-way ANOVA. ***C***, ***D***, lower panels: EPSC_16–20_ amplitude. ZnT3WT: 1.2 mm calcium: 996 pA ± 95 pA vs. 2.4 mm calcium: 1564 pA ± 135 pA, *n* = 5, *p* < 0.01, *F* = 58.51, DFn = 1, DFd = 40; ZnT3KO: 1.2 mm calcium: 750 pA ± 185 pA vs. 2.4 mm calcium: 876 pA ± 156 pA, *n* = 5, *p* = 0.24, *F* = 1.40, DFn = 1, DFd = 40; two-way ANOVA.

**Figure 3.**
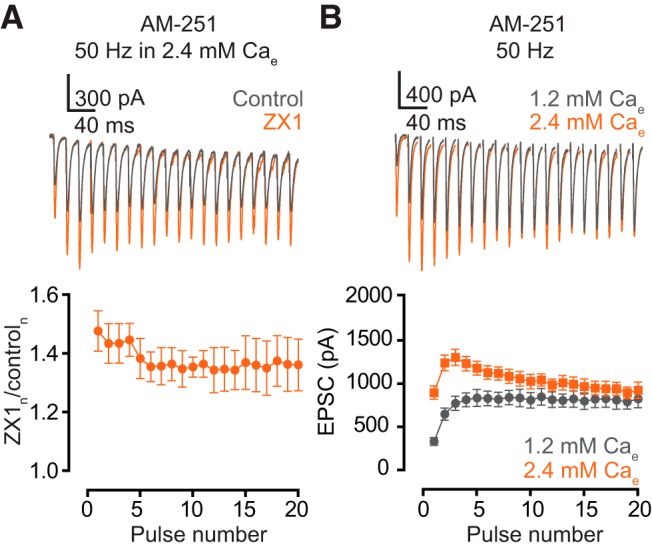
Endocannabinoid signaling is necessary for the zinc-mediated enhancement of EPSC_SS_. ***A***, Upper panel, representative traces of AMPA EPSCs, evoked by 50-Hz 20-pulse train, in the presence of 1 µm AM-251 before (gray) and after ZX1 (orange). Lower panel, summary graph showing average ratios of AMPA EPSCs in the presence of ZX1 to that in control during the train (ZX_1–2_/control_1–2_ vs. ZX_16–20_/control_16–20_: 50 Hz: *n* = 5, *p* = 0.13). ***B***, Upper panel, representative traces of AMPA EPSCs evoked by 50-Hz 20-pulse train in 1.2 mm (gray) and 2.4 mm (orange) external calcium in the presence of AM-251. Lower panel, summary graph showing average AMPA EPSC amplitudes during the train in 1.2 mm (gray) and 2.4 mm (orange) external calcium in the presence of AM-251. (EPSC_16–20_ amplitude in AM-251: 1.2 vs. 2.4 mm calcium, *n* = 5, *p* = 0. 09). Values represent mean ± SEM. Detailed values and statistical tests: ***A***, lower panel: ZX_1–2_/control_1–2_ vs. ZX_16–20_/control_16–20_: 50 Hz: control: 1.47 ± 0.06 vs. ZX1: 1.36 ± 0.08, *n* = 5, *p* = 0.13, *t* = 1.4, df = 9; paired *t*-test. ***B***, lower panel: EPSC_16–20_ amplitude: AM-251: 1.2 mm calcium: 805 pA ± 97 pA vs. 2.4 mm calcium: 921 pA ± 80 pA, *n* = 5, *p* = 0.19, *F* = 1.7, DFn = 1, DFd = 40; two-way ANOVA.

Brain slice preparation and electrophysiological experiments were carried out using extracellular solution artificial cerebrospinal fluid (ACSF) of the following composition (in mm): 130 NaCl, 3 KCl, 1.2 CaCl_2_·2H_2_O, 1.8 MgCl_2_⋅6H_2_O, 20 NaHCO_3_, 3 Hepes, and 10 d-glucose, saturated with 95% O_2_/5% CO_2_ (vol/vol), pH 7.25–7.35, ∼300 mOsm. To minimize changes in excitability of parallel fibers when switching ACSFs, divalent ion concentrations were optimized as follows: 1.2 mm external calcium ACSF consisted of 1.2 mm CaCl_2_ and 1.8 mm MgCl_2_, and 2.4 mm external calcium ACSF consisted of 2.4 mm CaCl_2_ and 0.6 mm MgCl_2_. Contaminating zinc was removed from the ACSF by stirring the ACSF with Chelex 100 resin (Bio-Rad) for 1 h. High-purity CaCl_2_⋅2H_2_O and MgCl_2_⋅6H_2_O salts (99.995% purity; Sigma-Aldrich) were added to the ACSF after the Chelex resin was filtered using Nalgene rapid-flow filters lined with polyethersulfone (0.2-µm pore size). All plastic and glassware used in these experiments were washed with 5% high-purity nitric acid. For brain slice preparations, mice were first anesthetized with isoflurane (3%) and then immediately decapitated. Brains were rapidly removed, and coronal slices (210 µm) of the left dorsal cochlear nucleus (DCN) were prepared in 1.2 mm external calcium containing ACSF at 35°C using a Vibratome (VT1200 S; Leica). Slices were then transferred to a holding chamber, where they were incubated for ∼60 min at 35°C. After incubation, slices were maintained at room temperature and used for experiments up to a duration of 4 h. For electrophysiological experiments, slices were transferred into the recording chamber and perfused with ACSF at a rate of 1–2 ml/min. Cartwheel cells in the molecular layer of the DCN were identified by their characteristic firing pattern that consists of simple and complex spikes (Zhang and Oertel, 1993; Manis et al., 1994; Tzounopoulos et al., 2004). Electrophysiological recordings were made using a MultiClamp-700B amplifier equipped with Digidata-1440A A/D converter (Molecular Devices). Whole-cell voltage-clamp recordings were conducted at –40 mV holding potential, except for Figs. 1 and 6, where neurons were held at –70 mV. For whole-cell recordings, we used borosilicate fire-polished glass pipettes with filament (Sutter Instruments). Recording pipettes were filled with a cesium-based internal solution with the following composition (in mm): 126 CsCH_3_O_3_S, 4 KCl, 10 Hepes, 4 Na_2_ATP, 0.3 Tris-GTP, 10 Tris-phosphocreatine, 1 Cs_2_EGTA, 1 QX-314, and 3 sodium ascorbate (pH 7.25, 295 mOsm), 3–5 MΩ resistance. Experimental results reported in Figs. 1 and 6 were conducted using a K-based internal solution containing (in mm) 113 K-gluconate, 4.5 MgCl_2_·6H_2_O, 14 Tris-phosphocreatine, 9 Hepes, 0.1 EGTA, 4 Na_2_ATP, 0.3 Tris-GTP, and 10 sucrose (pH 7.3, 300 mOsm). Recordings were conducted at 34–37°C using an inline heating system. Electrode series resistance was compensated at 70%–80% on-line using a 10-µs lag. Data were sampled at 10 or 20 kHz and filtered at 4 or 8 kHz. AMPA EPSCs in cartwheel cells were evoked by stimulating parallel fibers with an Isoflex stimulator (AMPI) using ACSF containing glass theta electrodes. AMPA EPSC peak amplitudes were measured from the baseline obtained by extrapolating the decay of the preceding AMPA EPSC using a single-exponential function. All experiments were conducted in the presence of glycine and GABA_A_ receptor blockers, strychnine (1 µm), and SR95531 (20 µm), respectively. To validate that NMDARs do not contribute to the zinc-mediated effects during the train, experiments in Fig. 2 and Fig. 3 were conducted in the presence of 50 µm of NMDAR antagonist, DL-APV. ZX1 (100 µm), AM-251 (1 µm), WIN 55, 212-2 (50 nm), cyclothiazide (100 µm), and kynurenic acid (0.5–1 mm) were always bath applied. All drugs were dissolved in deionized water, except for WIN 55, 212-2, which used DMSO as vehicle; the final concentration of DMSO was <0.5%. The experimenter was blinded to the genotype in experiments involving ZnT3WT and ZnT3KO mice. Once the experimenter completed the analysis of experiments from individual mice, he received information regarding the grouping of mice. After the experimenter averaged the data from the two groups of mice, he was informed on the genotype of the two groups.

### Drugs

SR95531, DL-APV, and strychnine were purchased from Hello-Bio. AM-251, WIN 55, 212-2, QX-314, cyclothiazide, and kynurenic acid were purchased from Tocris. ZX1 was purchased from STREM Chemicals.

### Statistics

**Figure 4.**
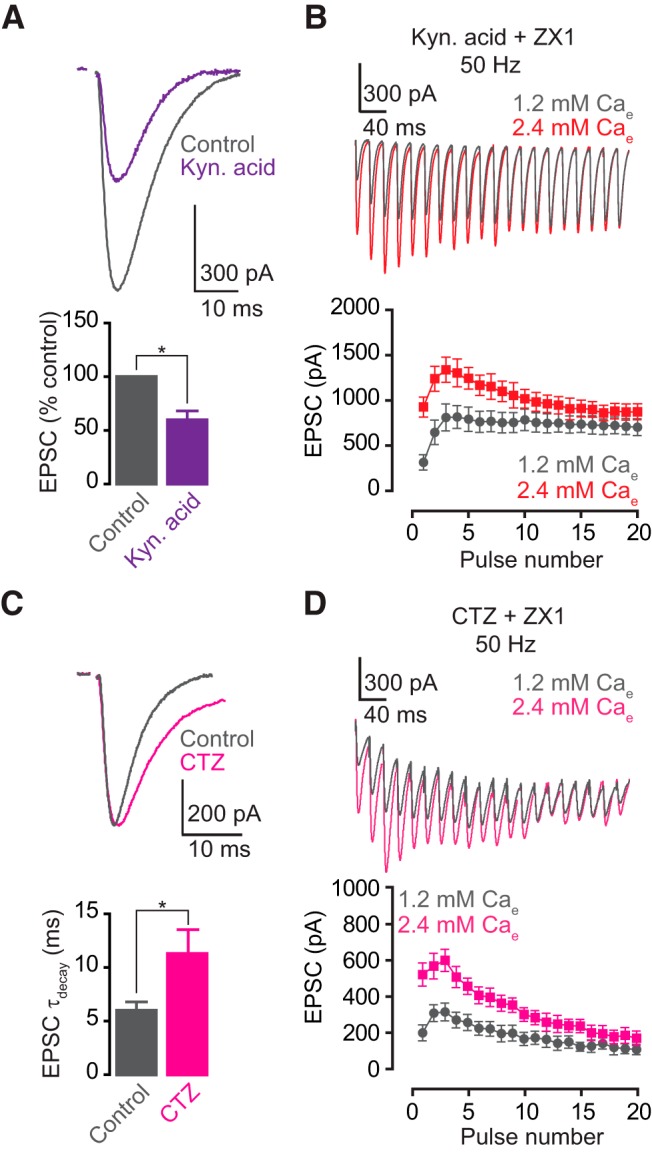
AMPAR saturation or desensitization do not contribute to the zinc-mediated enhancement of EPSC_SS_. ***A***, ***C***, Upper panels, representative traces of AMPA EPSCs before (gray) and after (color) bath application of 0.5–1 mm kynurenic acid (***A***) and 100 µm cyclothiazide (***C***). Lower panels, summary graphs showing normalized AMPA EPSC amplitude (***A***) and decay time constant (***C***), before (gray) and after (color) 0.5–1 mm kynurenic acid (***A***) and 100 µm cyclothiazide (***C***; AMPA EPSC amplitude: control vs. kynurenic acid, *n* = 5, *p* < 0.01; AMPA EPSC decay time constant: control vs. cyclothiazide, *n* = 5, *p* = 0.04). ***B***, ***D***, Upper panels, representative traces of AMPA EPSCs evoked by 50-Hz 20-pulse train in 1.2 mm (gray) and 2.4 mm (color) external calcium, in 0.5–1 mm kynurenic acid and ZX1 (***B***) or in 100 µm cyclothiazide and ZX1 (***D***). Lower panels, summary graphs showing average AMPA EPSC amplitudes during the train, in kynurenic acid and ZX1 (***B***), or in cyclothiazide and ZX1 (***D***). (EPSC_16–20_ amplitude: kynurenic acid: 1.2 vs. 2.4 mm calcium, *n* = 5, *p* = 0.13; cyclothiazide: 1.2 vs. 2.4 mm calcium, *n* = 5, *p* = 0.06.) Values represent mean ± SEM. Detailed values and statistical tests: ***A***, lower panel: AMPA EPSC amplitude in the presence of kynurenic acid: 59.63% ± 8.5% of baseline, *n* = 5, *p* < 0.01, *t* = 4.74, df = 4; one-sample *t*-test. ***C***, lower panel: AMPA EPSC decay time constant: control vs. cyclothiazide: 5.99 ± 0.81 vs. 11.32 ± 2.15, *n* = 5, *p* = 0.04; paired *t*-test, *t* = 2.95, df = 4. ***B***, lower panel: EPSC_16–20_ amplitude: kynurenic acid: 1.2 mm calcium: 718 pA ± 94 pA vs. 2.4 mm calcium: 812 pA ± 96 pA, *n* = 5, *p* = 0.13, *F* = 2.37, DFn = 1, DFd = 40; two-way ANOVA. ***D***, lower panel: cyclothiazide: 1.2 mm calcium: 122 pA ± 32 pA vs. 2.4 mm calcium: 186 pA ± 41 pA, *n* = 5, *p* = 0.06, *F* = 3.6, DFn = 1, DFd = 40; two-way ANOVA.

**Figure 5.**
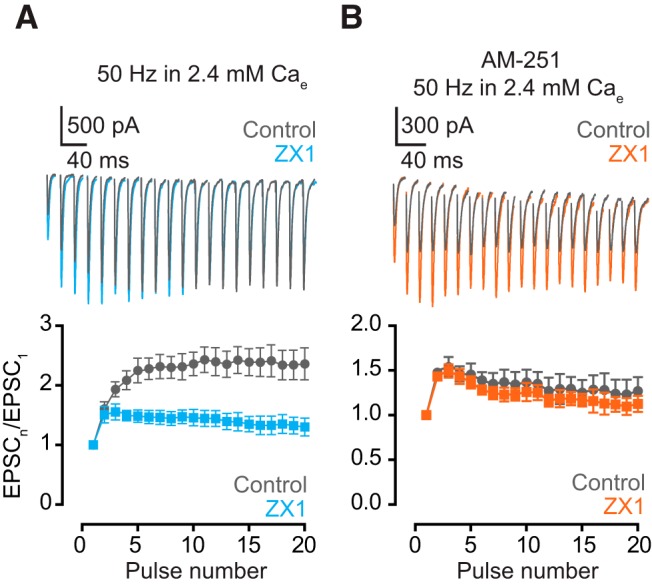
Enhanced zinc-mediated synaptic facilitation depends on endocannabinoid signaling. ***A***, ***B***, Upper panels, representative traces of AMPA EPSCs evoked by 50-Hz 20-pulse train before (gray) and after ZX1 application (color), in the absence (***A***) or presence (***B***) of AM-251. Lower panels, summary graphs showing average AMPA EPSC amplitudes during the train, normalized to EPSC amplitude elicited by first pulse, before (***A*** and ***B***, gray) and after (***A*** and ***B***, color) ZX1 application, in the absence (***A***), or presence (***B***) of AM-251 (ratio of the amplitude of 16–20 EPSC to first EPSC at 50 Hz: in the absence of AM-251: control vs. ZX1, *n* = 10, *p* < 0.01; in the presence of AM-251: control vs. ZX1, *n* = 5, *p* = 0.32). Values represent mean ± SEM. Detailed values and statistical tests: Lower panels: ratio of the amplitude of 16–20 EPSC to first EPSC at 50 Hz: in the absence of AM-251: control: 2.29 ± 0.25 vs. ZX1: 1.32 ± 0.15, *n* = 10, *p* < 0.01, *F* = 52.81, DFn = 1, DFd = 90; in the presence of AM-251: control: 1.19 ± 0.15 vs. ZX1: 1.04 ± 0.09, *n* = 5, *p* = 0.32, *F* = 1.0, DFn = 1, DFd = 40; two-way ANOVA.

**Figure 6.**
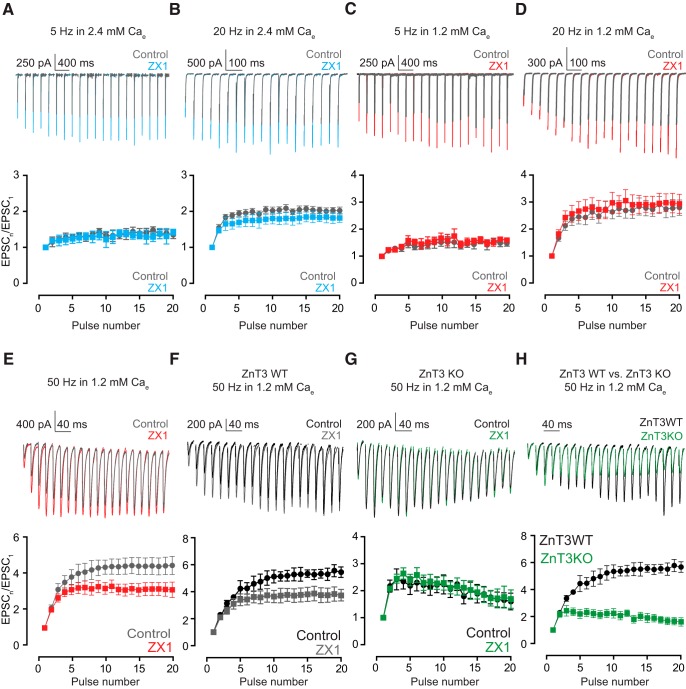
AMPA EPSC facilitation by synaptically released zinc is frequency, history, and Pr dependent. ***A***, ***B***, Upper panels, representative traces of AMPA EPSCs evoked by 20-pulse train at 5 Hz (***A***) and 20 Hz (***B***), before (gray) and after (color) 100 µM ZX1, in 2.4 mm external calcium. Lower panels, summary graphs showing average AMPA EPSC amplitudes during the 20-pulse train, normalized to EPSC amplitude of the first pulse, before (gray) and after (color) ZX1 application at 5 Hz (***A***) and 20 Hz (***B***), in 2.4 mm external calcium (ratio of the amplitude of 16–20 EPSC to first EPSC: 2.4 mm calcium: 5 Hz: control vs. ZX1, *n* = 5, *p* = 0.58; 20 Hz: control vs. ZX1, *n* = 5, *p* = 0.09). ***C–E***, Upper panels, representative traces of AMPA EPSCs evoked by 20-pulse train at 5 Hz (***C***), 20 Hz (***D***), and 50 Hz (***E***), before (gray) and after (color) 100 µM ZX1, in 1.2 mm external calcium. Lower panels, summary graphs showing average AMPA EPSC amplitudes during the 20-pulse train, normalized to EPSC amplitude of the first pulse, before (gray) and after (color) ZX1 application at 5 Hz (***C***), 20 Hz (***D***), and 50 Hz (***E***) in 1.2 mm external calcium (ratio of the amplitude of 16–20 EPSC to first EPSC: 1.2 mm calcium: 5 Hz: control vs. ZX1, *n* = 5, *p* = 0.34; 20 Hz: control vs. ZX1, *n* = 5, *p* = 0.45; 50 Hz vs. ZX1, *n* = 10, *p* < 0.01). ***F***, ***G***, Upper panels, representative traces of AMPA EPSCs evoked by 50-Hz 20-pulse train, before (black) and after (color) 100 µm ZX1, in ZnT3WT (***F***) and ZnT3KO (***G***) mice. Lower panels, summary graphs showing average AMPA EPSC amplitudes during the 50-Hz 20-pulse train, normalized to EPSC amplitude of the first pulse, before (black) and after (color) ZX1 application, in ZnT3WT (***F***) and ZnT3KO (***G***) mice (ratio of the amplitude of 16–20 EPSC to first EPSC: ZnT3WT mice: 50 Hz control vs. ZX1, *n* = 6, *p* < 0.01; ZnT3KO mice: 50 Hz: control vs. ZX1, *n* = 5, *p* = 0.85). ***H***, Upper panel, representative traces of peak-scaled AMPA EPSCs evoked by 50-Hz 20-pulse train in ZnT3WT (black) and ZnT3KO mice (green). Lower panel, summary graphs showing average AMPA EPSC amplitudes during the 50-Hz 20 pulse train, normalized to EPSC amplitude of the first pulse in control conditions, in ZnT3WT (black) and ZnT3KO (color) mice (ratio of the amplitude of 20 EPSC to first EPSC: ZnT3WT vs. ZnT3KO: control: 50 Hz, *n* = 5, *p* < 0.01). Values represent mean ± SEM. Detailed values and statistical tests: ***A***, ***B***, lower panels: ratio of the amplitude of 16–20 EPSC to first EPSC: 2.4 mm calcium: 5 Hz: control: 1.40 ± 0.12 vs. ZX1: 1.34 ± 0.15, *n* = 5, *p* = 0.58, *F* = 0.31, DFn = 1, DFd = 40; 20 Hz: control: 2.03 ± 0.09 vs. ZX1: 1.83 ± 0.14, *n* = 5, *p* = 0.09, *F* = 3.0, DFn = 1, DFd = 40; two-way ANOVA. ***C–E***, lower panels: ratio of the amplitude of 16–20 EPSC to first EPSC: 1.2 mm calcium: 5 Hz: control: 1.54 ± 0.14 vs. ZX1: 1.61 ± 0.11, *n* = 5, *p* = 0.34, *F* = 0.9, DFn = 1, DFd = 40; 20 Hz: control: 2.78 ± 0.29 vs. ZX1: 2.94 ± 0.34, *n* = 5, *p* = 0.45, *F* = 0.56, DFn = 1, DFd = 40; 50 Hz: 4.44 ± 0.47 vs. ZX1: 3.14 ± 0.37, *n* = 10, *p* < 0.01, *F* = 22.59, DFn = 1, DFd = 90; two-way ANOVA. ***F***, ***G***, lower panels: ratio of the amplitude of 16–20 EPSC to first EPSC: ZnT3WT mice: 50 Hz: 5.580 ± 0.39 vs. ZX1: 3.96 ± 0.55, *n* = 6, *p* < 0.01, *F* = 28.37, DFn = 1, DFd = 50; ZnT3KO mice: control: 50 Hz: 1.71 ± 0.27 vs. ZX1: 1.74 ± 0.33, *n* = 5, *p* = 0.85, *F* = 0.04, DFn = 1, DFd = 40; two-way ANOVA. ***H***, lower panel: ratio of the amplitude of 16–20 EPSC to first EPSC: ZnTWT vs. ZnT3KO: control: 50 Hz: 5.58 ± 0.39 vs. 1.71 ± 0.27, *n* = 5, *p* < 0.01, *t* = 8.15, df = 8; unpaired *t*-test.

**Figure 7.**
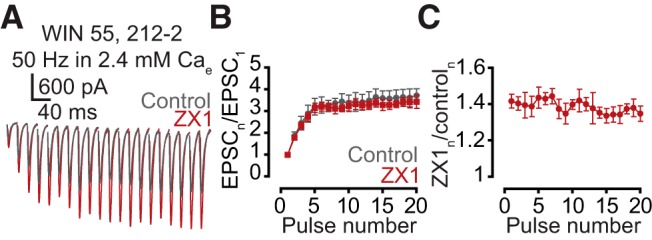
The postsynaptic inhibitory effect of zinc on AMPA EPSC does not contribute to the zinc-mediated enhancement of synaptic facilitation. ***A***, Representative traces of AMPA EPSCs, evoked by 50-Hz 20-pulse train in the presence of 50 nm WIN 55 212-2, before (gray) and after (maroon) ZX1. ***B***, Summary graph showing average AMPA EPSC amplitudes during the train, normalized to EPSC amplitude elicited by the first pulse, before (gray) and after (maroon) ZX1 (ratio of the amplitude of 16–20 EPSC to first EPSC at 50 Hz: control vs. ZX1, *n* = 5, *p* = 0.18). ***C***, Summary graph showing average ratios of AMPA EPSCs in the presence of ZX1 to that in control during the train (ZX_1–2_/control_1–2_ vs. ZX_16–20_/control_16–20,_ *n* = 5, *p* = 0.23). Values represent mean ± SEM. Detailed values and statistical tests: ***B***, Ratio of the amplitude of 16–20 EPSC to first EPSC at 50 Hz: control: 3.63 ± 0.35 vs. ZX1: 3.23 ± 0.22, *n* = 5, *p* = 0.18, *F* = 1.84, DFn = 1, DFd = 40; two-way ANOVA; ***C***, ZX_1–2_/control_1–2_ vs. ZX_16–20_/control_16–20_: 1.40 ± 0.04 vs. 1.35 ± 0.05, *n* = 5, *p* = 0.23, *t* = 1.39, df = 4, paired *t*-test.

For statistical comparisons, paired *t* tests and unpaired *t* tests were used if the group data passed the Lilliefors test for normality. If the group data were not normally distributed, then the Wilcoxon rank sum test was used. For normalized data, one-sample *t*-test was used. We used two-way ANOVA for comparisons of data groups including multiple Ca levels shown in Figs. 2, 3*B*, 4*B*, 4*D*, 5*A*, 5*B*, 6*A*, 6*G*, and 7*B*.

## Results

### Synaptically released zinc enhances steady-state AMPA EPSC during high Pr and high-frequency trains

To investigate the impact of synaptically released zinc in neurotransmission during different frequencies and levels of synaptic activity, we studied zinc-mediated neuromodulation in cartwheel cells. Cartwheel cells represent a class of inhibitory interneurons located in the molecular layer of the DCN, a cerebellar-like structure in the auditory brainstem (Oertel and Young, 2004). Cartwheel cells receive glutamatergic input from zinc-rich parallel fibers (PFs; Frederickson et al., 1988). In response to PF stimulation by a 20-pulse train at 5 or 20 Hz, zinc chelation with the extracellular, high-affinity, fast chelator, ZX1 (Pan et al., 2011; Anderson et al., 2015), potentiated AMPA EPSC amplitudes (Fig. 1*A*, *B*). Moreover, the amount of this increase was constant throughout the stimulus train (Fig. 1*A*, *B*, *D*, *E*). This result suggests that the effect of zinc is a steady subtractive inhibition of AMPA EPSC, readily predicted by the postsynaptic inhibitory effect of zinc on AMPARs in response to a single stimulus (Kalappa et al., 2015). In contrast to these findings, during the 20-pulse stimulation at 50 Hz, ZX1 enhanced AMPA EPSC in the first two to four pulses but, paradoxically, inhibited steady-state responses (EPSC_SS_) during the last four pulses (Fig. 1*C–E*). This result suggests that the modulatory effects of zinc depend on the history of the synapse: during high-frequency stimulation, zinc inhibits EPSCs for the first few stimuli, but later in the stimulus train, it enhances EPSC_SS_. Given the known inhibitory role of zinc on synaptic strength, the zinc-mediated enhancement of EPSC_SS_ was unexpected.

The zinc-dependent enhancing effect on EPSC_SS_ was observed during high-frequency stimulation, which is associated with increases in Pr. To further interrogate the influence of Pr on EPSC_SS_ amplitudes, we manipulated Pr by using different extracellular calcium concentrations (Ca_e_). Within the same cell, increasing Ca_e_ from 1.2 mm (low Pr) to 2.4 mm (high Pr) resulted in an increase in EPSC_SS_ amplitude (Fig. 2*A*). Previous studies on the homologous cerebellar PF synapses onto Purkinje neurons, which lack synaptic zinc, showed that alterations in Pr do not affect EPSC_SS_ amplitudes during 50-Hz trains (Kreitzer and Regehr, 2000). Because zinc enhances AMPA EPSC_SS_ in DCN synapses during high-frequency trains (Fig. 1*C*, *E*), we hypothesized that zinc signaling is necessary for the increase in steady-state responses in higher Ca_e_. To test this hypothesis, we conducted similar experiments as in Fig. 2*A* but in the presence of ZX1. In these experiments, stimulation of PFs using the same 20-pulse 50-Hz train resulted in AMPAR EPSC_SS_ amplitudes that were not different between high and low Pr conditions (Fig. 2*B*). These results suggest that during high-frequency stimulation, endogenous zinc enhances EPSC_SS_ in higher Pr.

To determine whether synaptically released zinc was required for the enhanced EPSC_SS_ during high-frequency stimulation, we investigated EPSCss in PF to cartwheel cell synapses from mice lacking the ZnT3 transporter (ZnT3KO mice), which lack synaptically released zinc throughout the brain (Cole et al., 1999). In ZnT3KO mice, EPSC_SS_ evoked by a 20-pulse train stimulus at 50 Hz did not differ between 1.2 and 2.4 mm Ca_e_ (Fig. 2*D*), but EPSC_SS_ in ZnT3WT mice were larger in 2.4 mm Ca_e_ (Fig. 2*C*). Given that tonic zinc levels in the DCN are ZnT3 independent (Anderson et al., 2015), these results suggest that ZnT3-dependent synaptically released zinc enhances EPSC_SS_ in high Pr.

### Synaptic zinc–mediated endocannabinoid signaling enhances steady-state AMPAR EPSC by enhancing synaptic facilitation

Zinc triggers endocannabinoid release after trains of presynaptic action potentials (Perez-Rosello et al., 2013). We therefore tested whether endocannabinoid signaling is necessary for the zinc-dependent enhancement of EPSC_SS_. In the presence of 1 µm AM-251, a cannabinoid receptor 1 specific antagonist (Lan et al., 1999), we did not observe any change in the amount of ZX1 potentiation throughout the 50-Hz stimulation (Fig. 3*A*). Moreover, in ZX1, EPSC_SS_ were not different between 1.2 and 2.4 mm Ca_e_ (Fig. 3*B*). These results suggest that endocannabinoid signaling is necessary for the zinc-dependent enhancement of EPSC_SS_.

By reducing Pr (Kreitzer and Regehr, 2002; Wilson and Nicoll, 2002), endocannabinoid signaling may also reduce the effect of postsynaptic factors that inhibit EPSC_SS_, such as receptor saturation and desensitization (Trussell et al., 1993; Wadiche and Jahr, 2001; Chen et al., 2002; Foster et al., 2002). Alternatively, endocannabinoid signaling may enhance presynaptic factors that also enhance EPSC_SS_, such as synaptic facilitation (Zucker and Regehr, 2002).

First we investigated whether reductions in AMPAR saturation or desensitization contribute to enhanced EPSC_SS_. Namely, in ZX1, we measured the effect of kynurenic acid (0.5–1 mm) or cyclothiazide (100 µm) in EPSC_SS_, with either 1.2 or 2.4 mm Ca_e_. Kynurenic acid is a competitive AMPAR antagonist (Fig. 4*A*) that inhibits receptor saturation, and cyclothiazide is an allosteric modulator (Fig. 4*C*) that inhibits AMPAR desensitization (Partin et al., 1994, 1996; Neher and Sakaba, 2001; Prescott et al., 2006). If the enhancing effect of zinc on EPSC_SS_ was mediated by a zinc-driven decrease in AMPAR saturation or desensitization, blockade of these processes would enhance EPSC_SS_ in high Pr even in the presence of ZX1. Under these conditions, high Pr did not enhance EPSC_SS_ (Fig. 4*B*, *D*), suggesting that the enhancing effect of zinc on EPSC_SS_ is not due to either AMPAR saturation or desensitization.

Next we investigated whether endogenous zinc enhances EPSC_SS_ by endocannabinoid-mediated Pr reduction and a subsequent enhancement of synaptic facilitation. Consistent with this hypothesis, ZX1 decreased AMPA EPSC facilitation at 50 Hz (Fig. 5*A*). In contrast, in the presence of AM-251, ZX1 did not decrease AMPA EPSC facilitation (Fig. 5*B*), suggesting that endocannabinoid signaling and enhanced synaptic facilitation are necessary for the zinc-mediated augmentation of EPSC_SS_.

Consistent with the lack of effect of ZX1 in enhancing EPSC_SS_ at lower stimulation frequencies (Fig. 1*A*, *B*, *D*, *E*), ZX1 did not affect synaptic facilitation at these frequencies in either 1.2 or 2.4 mm Ca_e_ (Fig. 6*A–D*). In contrast, ZX1 decreased synaptic facilitation at 50 Hz in 1.2 mm Ca_e_. However, ZX1 decreased synaptic facilitation by the eighth pulse in 1.2 mm Ca_e_, but in 2.4 mm Ca_e_ it decreased facilitation by the third pulse (Fig. 5*A* vs. Fig. 6*E*). This result is consistent with the more robust zinc-dependent endocannabinoid signaling occurring at higher Pr, which is reflected by the requirement for fewer stimuli to elicit endocannabinoid release in higher Pr (Perez-Rosello et al., 2013). Finally, ZX1 did not change synaptic facilitation at 50 Hz in ZnT3KO mice (Fig. 6*F–H*). Together, these results suggest that synaptically released zinc results in increased synaptic facilitation and enhanced EPSC_SS_, via retrograde endocannabinoid signaling and decreased Pr.

Elimination of the postsynaptic inhibitory effect of zinc on AMPA EPSC amplitude during the 50-Hz train could also contribute to the zinc-mediated enhancement of synaptic facilitation. To explore this possibility, we occluded the endocannabinoid-mediated effect on synaptic facilitation by applying 50 nm WIN 55, 212-2 (WIN), a CB1 receptor activator. Then, we tested whether this occlusion affected the ZX1 potentiation of the EPSC throughout the train. If a potential reduction of the postsynaptic inhibitory effect of zinc on AMPA EPSC amplitude during the 50-Hz train was contributing to the zinc-mediated enhancement of synaptic facilitation, then occlusion of synaptic facilitation with WIN would also show a gradual reduction in the ZX1 potentiation throughout the train, as in Fig. 1*D*. We found that WIN occluded the effect of ZX1 on synaptic facilitation (Fig. 7*A*, *B*), and there was no reduction in the amount of ZX1 potentiation throughout the 50-Hz train (Fig. 7*A*, *C*). This result suggests that changes in the magnitude of the postsynaptic inhibitory effect of zinc on AMPA EPSC do not contribute to the zinc-mediated enhancement of synaptic facilitation.

## Discussion

Our results indicate that the effects of synaptically released zinc on excitatory neurotransmission are not purely inhibitory as previously thought, for zinc’s actions depend on the level of activity of the target synapses. For synapses that are active at high rates of presynaptic activity, synaptically released zinc leads to an initial inhibition of postsynaptic activity, likely via direct inhibition of AMPARs (Kalappa et al., 2015), followed by the recruitment of endocannabinoid signaling that leads to reduced Pr but enhanced steady-state EPSCs. This seemingly paradoxical effect is likely explained by the fact that increased Pr depletes presynaptic vesicles, saturates release, and limits the extent of facilitation (Zucker and Regehr, 2002; Jackman et al., 2016). Whereas our findings are consistent with this hypothesis, we cannot rule out potential effects of presynaptic endocannabinoid signaling on the presynaptic calcium signal that induces facilitation or on the calcium sensor synaptotagmin 7, which is required for synaptic facilitation (Jackman et al., 2016).

Facilitating synapses favor the transmission of burst-like presynaptic stimuli (Lisman, 1997; Abbott and Regehr, 2004). Whereas *in vivo* recordings from DCN granule cells have not been obtained, sensory stimulation produces bursts of high-frequency (>200 Hz) spikes in cerebellar granule cells (Chadderton et al., 2004). Although these bursts are briefer than the trains we used here, they also occur at higher frequencies. Based on our results showing that fewer stimuli are needed to elicit endocannabinoid signaling during higher stimulation frequencies (compare Fig. 5*A* and Fig. 6*E*), we expect that higher stimulation frequencies would also require fewer stimuli for eliciting the enhancing effect of zinc on steady-state EPSCs. Therefore, given the strong resemblance between DCN and cerebellar granule cells (Oertel and Young, 2004), we hypothesize that the effect of zinc in maintaining enhanced synaptic facilitation during bursts of activity may enhance information flow in cartwheel cells. However, because our *in vitro* stimulation may be stronger than the *in vivo* pattern of activity, further *in vivo* studies using natural stimuli are required to determine how synaptic zinc affects information coding in the molecular layer of the DCN.

We previously reported that synaptically released zinc decreases post-tetanic potentiation induced by high-frequency stimulation (Perez-Rosello et al., 2013); however, the effect of zinc during the high-frequency stimulation had not been studied. By assessing the role of synaptically released zinc during low- and high-frequency trains, we found that synaptic strength at intensely active synapses is larger under conditions that inhibit Pr, such as zinc-dependent endocannabinoid signaling. These results complement and extend previous studies on presynaptic GABA_B_ receptors, which sustain neurotransmission at higher stimulus frequencies by reducing Pr and, in turn, controlling AMPAR desensitization (Brenowitz et al., 1998; Brenowitz and Trussell, 2001). Whereas zinc and GABA_B_ receptors exert their context-dependent effects via different mechanisms, a common concept emerges supporting that neuromodulatory systems that combine pre- and postsynaptic mechanisms of actions also display a unique flexibility in mediating bidirectional modulation of EPSC strength (Tzounopoulos et al., 2007).

Our findings on zinc-mediated promotion of endocannabinoid signaling are consistent with our previous report showing that during high-frequency stimulation synaptic zinc release, activation of GPR39 receptors and subsequent rises in postsynaptic calcium and phospholipase C activation are necessary for triggering the synthesis of the endocannabinoid 2-arachidonoylglycerol in PF DCN synapses (Perez-Rosello et al., 2013). Moreover, the lack of zinc-dependent enhancement in EPSC_SS_ during lower stimulation frequencies, as well as the development of the endocannabinoid-mediated effect later in the train during high-frequency stimulation, are consistent with the notion that endocannabinoid signaling in DCN synapses is recruited in response to sustained high-frequency bursts of neuronal activity (Sedlacek et al., 2011; Perez-Rosello et al., 2013).

Prior studies have reported a lack of effect of synaptic zinc on short-term synaptic plasticity of AMPA EPSCs in zinc-rich hippocampal mossy fiber to CA3 (MF-CA3) or Schaffer collateral to CA1 (SC-CA1) synapses (Vogt et al., 2000; Vergnano et al., 2014). The lack of effect in these studies could be because the investigated synapses lack or express weak endocannabinoid signaling (Ohno-Shosaku et al., 2002; Hofmann et al., 2008). Moreover, these studies used a narrower range of stimulation frequencies that did not exceed 25 Hz. This could also explain the lack of effect of zinc on short-term plasticity, because higher or more sustained stimulation frequencies are required for the recruitment of endocannabinoid signaling (Perez-Rosello et al., 2013), which, in turn, is crucial for the effects of zinc on short-term plasticity of glutamatergic neurotransmission. Finally, although these studies used ZnT3KO mice to eliminate synaptic zinc, zinc was chelated with tricine or CaEDTA. However, CaEDTA has slower kinetics for zinc binding than ZX1, and tricine has weaker zinc-binding affinity than ZX1 (Anderson et al., 2015). Therefore, CaEDTA and tricine are less efficient than ZX1 for investigating the effects of fast, transient elevations of synaptic zinc on synaptic targets, such as NMDAR and AMPARs (Anderson et al., 2015; Kalappa et al., 2015). Together, these data suggest that the context-dependent effect of zinc on glutamatergic neurotransmission is synapse specific and, like the effects of other neuromodulatory systems, depends on the molecular composition, structure, and function of different synapses.

Recent studies explored the dynamics of zinc in the synaptic cleft and extracellular space (Vergnano et al., 2014; Anderson et al., 2015), but less is known about the presynaptic zinc dynamics. Whereas one study suggests that zinc-containing vesicles predominantly dominate the reserve pool of synaptic vesicles (Lavoie et al., 2011), the exocytosis and endocytosis dynamics of zinc-containing vesicles remain unknown. Our results are inconsistent with depletion of zinc-containing vesicles during the high-frequency train, for ZX1 revealed no change in the amount of AMPA EPSC potentiation throughout the train of stimuli in the presence of exogenously applied cannabinoids (Fig. 7). This conclusion is based on the assumption that zinc-mediated AMPA EPSC inhibition tracks synaptic zinc levels in the cleft. However, recent results proposed AMPA EPSC modulation via intracellular zinc signaling in cultured hippocampal neurons (Arons et al., 2016). As such, transient increases in extracellular zinc after synaptic stimulation could lead to translocation of zinc into the postsynaptic neuron via zinc-permeable AMPARs (Jia et al., 2002), inducing, in turn, changes in intracellular zinc levels and modulation of AMPA EPSCs that does not track extracellular zinc levels. NMDARs contain extracellular high-affinity binding sites capable of tracking extracellular zinc levels (Paoletti et al., 2009; Hansen et al., 2014), and therefore future studies assessing the effect of synaptic zinc on NMDARs, or using localized extracellular zinc-sensitive dyes to quantify zinc levels during trains of presynaptic activity, are needed for elucidating the time course of exocytosis and endocytosis of zinc-containing vesicles.

In summary, this work demonstrates that during high-frequency trains of presynaptic activity, synaptic zinc scales glutamatergic neurotransmission bidirectionally. During the onset of activity, glutamatergic neurotransmission is scaled down predominantly by postsynaptic zinc inhibition; however, later in the train, zinc-dependent endocannabinoid signaling decreases Pr, which, in turn, increases synaptic facilitation. This enhancement overrides the Pr decrease and postsynaptic zinc inhibitory effects, thus resulting in overall enhanced synaptic strength.

## Synthesis

Reviewing Editor: Pablo Castillo, Albert Einstein College of Medicine Decisions are customarily a result of the Reviewing Editor and the peer reviewers coming together and discussing their recommendations until a consensus is reached. When revisions are invited, a fact-based synthesis statement explaining their decision and outlining what is needed to prepare a revision will be listed below. The following reviewer(s) agreed to reveal their identity: Wade Regehr

Your manuscript has been evaluated by two reviewers. They have a good agreement on the scientific merit and quality of the work as it provides novel insights into zinc regulation of synaptic transmission at a particular central synapse. As described below, one reviewer would like you to town down the functional impact of your findings. There are also some minor comments that you should address before the final decision.

REVIEWER #1:

The authors have made significant changes to the manuscript that have addressed many of the prior concerns. The work is clearer and more fully reported now.

P14, l12: Just because you enhance the EPSP/Cs does not mean that you have enhanced "information coding". Indeed, the effects shown are associated with relatively strong and synchronous stimulation of the PFs. Currents of 1-1.5 nA for fibers that supply only a few endings with 40pA quantal size (Gardner et al., 1999) indicate release from of up to 25 PFs at a time and likely activation of at least 2-3x more synapses given the likely baseline Pr, and even more PFs given the difference in holding potentials between the present study and Gardner et al., 1999, which would cause the quantal size to be smaller in the present study. Thus, the stimulation is probably stronger, due to it's synchrony, than the in vivo pattern of activity, and may overemphasize the roles of both zinc and retrograde signalling mechanisms, which would be expected to be much more weakly activated with asynchronous granule cell activity. The experimental results remain very interesting in terms of dissecting mechanisms and thinking about the potential roles for this class of synaptic modulation, but it remains unclear from the way the experiments were done as to how "information coding" is really affected. In other words, I would tone this down a good deal. Also, does the change in synaptic dynamics affect "information coding" in the granule cells (as stated), or in the cartwheel cells? I would say the cartwheel cells, as the effects are likely dependent on the target cell. It would be worthwhile to include some caveats in the interpretation of the results until such time as experiments using much weaker, naturalistic stimuli are feasible and other target cell types have been more thoroughly investigated.

Minor:

P7, l20: "was" instead of "would" reads better here (the rest of the description is in past tense).

P12, l10: "for less number of stimuli" -< "for fewer stimuli"

P14, l5: "more brief" -< "briefer"

P14, l6 and l8: "less stimuli" -< "fewer stimuli"

P19, l18, 19, 20: the "p" is missing in front of the p-values here - should be added to remove any ambiguity.

Figures: Although the authors state that they have included the t-value and d.f. in the figure legends where statistics are reported, these values appear to be inconsistently reported (Figure 1 does have the values, but Figures 3-7 are not consistent, and ANOVA's should also report the F value and d.f.).

REVIEWER #2:

The authors have responded adequately to the reviews. The manuscript is of good quality and should get good exposure as an eNeuro paper.
